# Metabolic recovery from heavy exertion following banana compared to sugar beverage or water only ingestion: A randomized, crossover trial

**DOI:** 10.1371/journal.pone.0194843

**Published:** 2018-03-22

**Authors:** David C. Nieman, Nicholas D. Gillitt, Wei Sha, Debora Esposito, Sivapriya Ramamoorthy

**Affiliations:** 1 Human Performance Laboratory, Appalachian State University, North Carolina Research Campus, Kannapolis, North Carolina, United States of America; 2 Dole Nutrition Research Laboratory, North Carolina Research Campus, Kannapolis, North Carolina, United States of America; 3 Bioinformatics Services Division, University of North Carolina at Charlotte, North Carolina Research Campus, Kannapolis, North Carolina, United States of America; 4 Plants for Human Health Institute, North Carolina State University, North Carolina Research Campus, Kannapolis, North Carolina, United States of America; 5 Metabolon, Inc., Durham, North Carolina, United States of America; Weill Cornell Medical College Qatar, QATAR

## Abstract

**Objectives and methods:**

Using a randomized, crossover, counterbalanced approach, cyclists (N = 20, overnight fasted state) engaged in the four 75-km time trials (2-week washout) while ingesting two types of bananas with similar carbohydrate (CHO) but different phenolic content (Cavendish, CAV; mini-yellow, MIY, 63% higher polyphenols), a 6% sugar beverage (SUG), and water only (WAT). CHO intake was set at 0.2 g/kg every 15 minutes. Blood samples were collected pre-exercise and 0 h-, 0.75 h-,1.5 h-, 3 h-, 4.5 h-, 21 h-, 45 h-post-exercise.

**Results:**

Each of the CHO trials (CAV, MIY, SUG) compared to water was associated with higher post-exercise plasma glucose and fructose, and lower leukocyte counts, plasma 9+13 HODES, and IL-6, IL-10, and IL-1ra. OPLS-DA analysis showed that metabolic perturbation (N = 1,605 metabolites) for WAT (86.8±4.0 arbitrary units) was significantly greater and sustained than for CAV (70.4±3.9, P = 0.006), MIY (68.3±4.0, P = 0.002), and SUG (68.1±4.2, P = 0.002). VIP ranking (<3.0, N = 25 metabolites) showed that both CAV and MIY were associated with significant fold changes in metabolites including those from amino acid and xenobiotics pathways. OPLS-DA analysis of immediate post-exercise metabolite shifts showed a significant separation of CAV and MIY from both WAT and SUG (R2Y = 0.848, Q2Y = 0.409). COX-2 mRNA expression was lower in both CAV and MIY, but not SUG, versus WAT at 21-h post-exercise in THP-1 monocytes cultured in plasma samples. Analysis of immediate post-exercise samples showed a decrease in LPS-stimulated THP-1 monocyte extracellular acidification rate (ECAR) in CAV and MIY, but not SUG, compared to WAT.

**Conclusions:**

CHO ingestion from bananas or a sugar beverage had a comparable influence in attenuating metabolic perturbation and inflammation following 75-km cycling. Ex-vivo analysis with THP-1 monocytes supported a decrease in COX-2 mRNA expression and reduced reliance on glycolysis for ATP production following ingestion of bananas but not sugar water when compared to water alone.

**Trial registration:**

ClinicalTrials.gov, U.S. National Institutes of Health, identifier: NCT02994628

## Introduction

Bananas are the leading fruit produced and consumed globally, and are an important source of carbohydrate energy, potassium, vitamin B6, vitamin C, and other micronutrients. Of the hundreds of different varieties that exist around the world, the Cavendish banana is most widely consumed and exported, and provides 13.8 g sugars/100 g (1.9 g glucose, 2.2 g fructose, 9.7 g sucrose). This banana also contains a unique blend of secondary metabolites such as phenolics (7 mg/100 g fresh pulp), carotenoid compounds (73 μg/100 g), and catecholamines including dopamine (9.1 mg/100 g) and serotonin (2.8 mg/100 g) [1–6].

In a prior study, we compared the acute effect of ingesting Cavendish bananas (with water) versus a 6% carbohydrate drink (both providing 0.8 g/kg carbohydrate per hour) on 75-km cycling performance and post-exercise inflammation, oxidative stress, and immune biomarkers using metabolomics-based profiling [7]. Blood glucose levels and performance did not differ between the banana and 6% carbohydrate trials, exercise-induced increases in inflammation were similar and below levels previously measured during water-only studies in our lab. Aside from higher dopamine during the banana trial, metabolite shifts following 75-km cycling were not statistically different indicating a similar pattern of fuel substrate utilization. In a second metabolomics-based study, Cavendish banana compared to water ingestion before and during 75-km cycling provided carbohydrates (0.6 g/kg), catecholamines, and phenolics compatible with enhanced performance (5%), diminished inflammation, elevated antioxidant capacity, and decreased fatty acid mobilization and oxidation [8].

The mini-yellow banana [Musa acuminata AA ‘Lady Finger’ or Pisang mas] has a higher sugar (5.4%) and phenolic (63%) content than the Cavendish banana (see Methods section). We hypothesized that metabolite shifts following 75-km cycling would differ in cyclists ingesting Cavendish or mini-yellow bananas in comparison to water only or a 6% carbohydrate only beverage, and that this may impact post-exercise recovery from physiological stress.

The purpose of this study was to compare ingestion of the higher phenolic mini-yellow banana with the Cavendish banana, a 6% carb beverage (with the same sugar profile as the Cavendish banana), and water-only on metabolite shifts (using global metabolomics), oxidative stress, muscle damage, and inflammation following a 75-km cycling time trial. Emphasis was placed on multiple recovery samples (0 h-, 0.75 h-,1.5 h-, 3 h-, 4.5 h-, 6 h-, 21 h-, and 45 h-post-exercise) to enhance interpretation of recovery patterns. Ex-vivo plasma cultures with THP-1 monocytes were tested for cyclooxygenase-2 messenger ribonucleic acid (COX-2 mRNA) expression and real-time measurements of oxygen consumption rate (OCR) and extracellular acidification rate (ECAR) to determine if increases in plasma levels of banana-related metabolites following acute banana ingestion conferred any metabolic post-exercise advantage beyond those linked to carbohydrate intake.

## Materials and methods

The protocol for this trial and supporting Consolidated Standards of Reporting Trials (CONSORT) checklist are available as S1 Protocol and S1 Checklist.

### Participants

Participants included 20 male and female cyclists (ages 22–50 years) who regularly competed in road races (category 1 to 5) and were capable of cycling 75-km at race pace. During the 10-week period when data were being collected, participants maintained their typical training regimen, and avoided the use of vitamin and mineral supplements, herbs, and medications. Participants signed informed consent and study procedures were approved (24 February 2016, with closure on 11 November 2016) by the Institutional Review Board at Appalachian State University. Data were collected at the Human Performance Laboratory at the North Carolina Research Campus in Kannapolis, NC. The study was first submitted to ClinicalTrials.gov on November 24, 2015, but due to a communication error between the IRB office and the primary investigator, the ClinicalTrials.gov submission was not corrected, approved, and posted until December 16, 2016. The authors confirm that all ongoing and related trials for this intervention are registered.

### Research design

**Fig 1:**
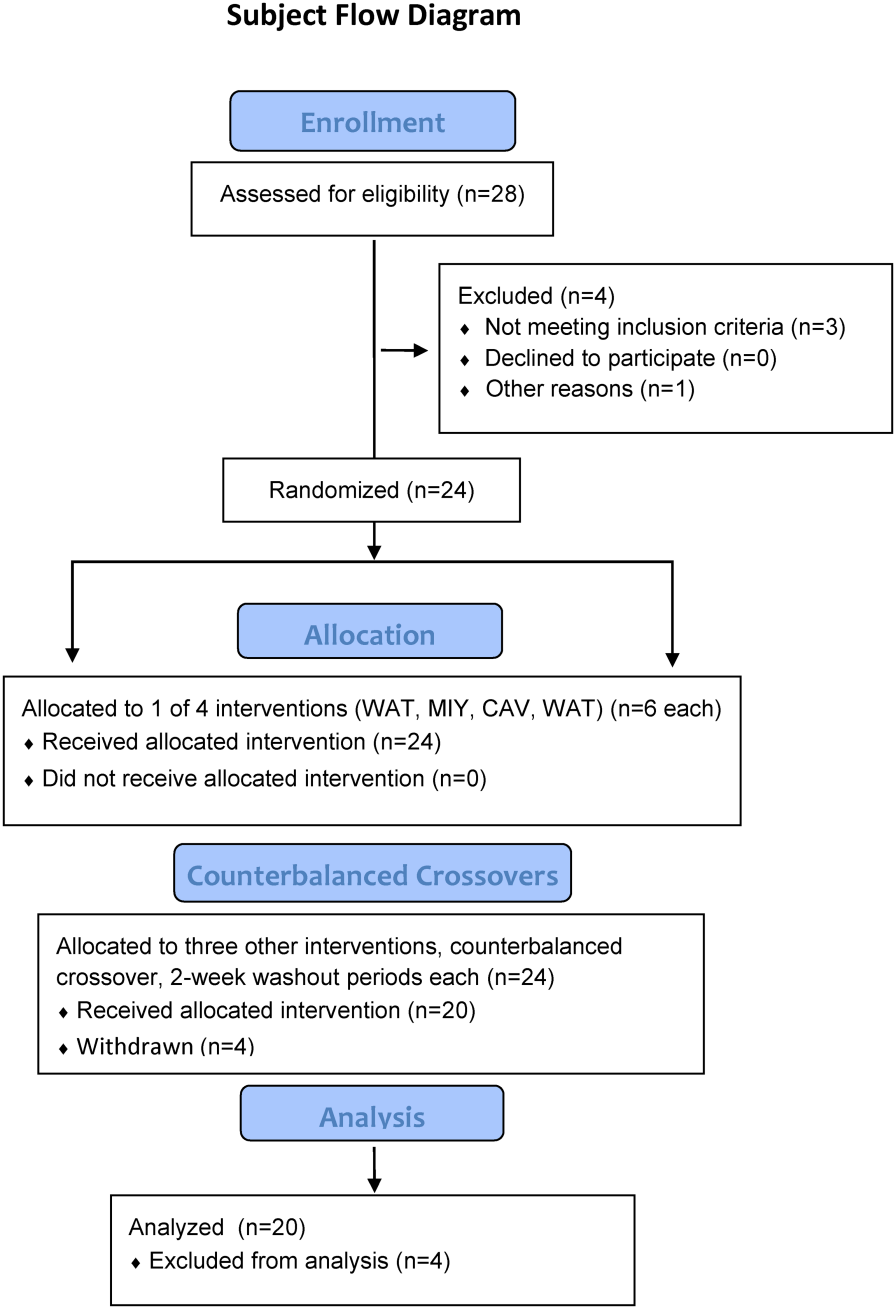
Subject flow diagram.

This study utilized a randomized (1:1 allocation, random number generator), crossover approach, and participants engaged in four 75-km cycling time trials while ingesting water only, Cavendish bananas, mini-yellow bananas, and a 6% carbohydrate beverage, separated by two weeks each (no blinding) (Fig 1). Participants completed the four arms of the study, and data were analyzed with participants operating as their own controls. Data were analyzed from subjects (N = 20) completing all aspects of the study using a repeated measures analysis of variance (ANOVA), within subject’s approach. Four subjects randomized into the study failed to complete all four arms of the study (three due to changes in personal schedules and one to a training-related injury).

One to two weeks prior to the first 75-km time trial, athletes completed orientation and baseline testing. Demographic and training histories were acquired with questionnaires. Maximal power, oxygen consumption, ventilation, and heart rate were measured during a graded exercise test (25 watts increase every two minutes, starting at 150 Watts) with the Cosmed Quark CPET metabolic cart (Rome, Italy) and the Lode cycle ergometer (Lode Excaliber Sport, Lode B.V., Groningen, Netherlands). Body composition was measured with the Bod Pod body composition analyzer (Life Measurement, Concord, CA).

During the 3-day period prior to each 75-km cycling trial, participants were asked to reduce the volume of their exercise training as if preparing for a race, and ingest a moderate-carbohydrate diet using a food list restricting high fat foods. Participants were instructed to keep the food record current by listing items immediately after they were eaten, to measure and record the volume using household measures (tablespoons, cups, slices, ounces), to provide sufficient detail about the method of preparation and include condiments, sugar, oils, butter, and other visible fats, and to avoid the tendency to eat less or under-report because of the recording process. The 3-day food records were analyzed for nutrient and flavonoid content using the Food Processor v. 11.1 (ESHA Research, Salem, OR). ESHA’s port utility (v. 4.0) was used to upload the Flavonoid Values for USDA Survey Foods and Beverages (FNDDS) 2007–2010 database [9], and each food/beverage was assessed for macro- and micro-nutrients, and total flavonoids.

For each of the four 75-km cycling trials, participants reported to the Human Performance Laboratory at 6:45 am in an overnight fasted state (no food or beverages other than water for at least 9 hours), and provided a pre-exercise blood sample. In accordance with the randomized schedule, participants then ingested 5 ml/kg water only, or water with 0.4 g/kg carbohydrate from Cavendish bananas or mini-yellow bananas (ripeness stage 5 or 6), or the 6% sugar beverage. The volume of mini-yellow banana consumed was adjusted for the 5.4% higher sugar content, and the 6% sugar beverage was formulated with the same ratio of sucrose, fructose, and glucose (2:1:1) measured in freeze-dried Cavendish bananas pre-, mid- and post-study (Ultra-High Performance Liquid Chromatography, Refractive Index Detection, Agilent 1200 series, Santa Clara, CA). Total phenolic content of freeze-dried Cavendish and mini-yellow bananas was determined using the Folin-Ciocalteu method [10].

After a 20-minute rest, participants warmed up and then began the 75-km cycling time trial using their own bicycles on CompuTrainer Pro Model 8001 trainers (RacerMate, Seattle, WA). The CompuTrainer MultiRider software system (version 3.0, RacerMate, Seattle, WA) was used to simulate a moderately difficult, mountainous 75-km course with continuous workload monitoring. Heart rate and rating of perceived exertion (RPE) were recorded every 30 minutes. Oxygen consumption and ventilation were measured using the Cosmed Quark CPET metabolic cart after 16 km and 55 km cycling (level sections of the course). Participants consumed 3 ml/kg water every 15 min, or water with 0.2 g/kg carbohydrate from one of the two banana types or the 6% sugar beverage every 15 minutes. No other beverages or food were allowed during the cycling time trials and 1.5-h recovery. Blood samples were taken via venipuncture immediately after and 0.75 h-,1.5 h-, 3 h-, 4.5 h-, 21 h-, 45 h-post-exercise after completing each of the 75-km time trial. The 21 h- and 45 h-post-exercise samples were obtained from participants at ~7:00 am in an overnight fasted state. All blood samples were centrifuged, aliquoted, and stored at -80°C until analysis. The four trials were separated by two weeks, after which participants crossed over to the next randomized condition, and repeated all procedures.

Participants provided responses to a symptom questionnaire within 10 minutes of completing each of the 75-km cycling trials [7,8]. The symptom questionnaire included questions on digestive health (feeling full, bloating, diarrhea, and nausea/vomiting), energy levels, focus/concentration, muscle cramping, and overall well-being. Subjects indicated responses using a 12- point Likert scale, with 1 relating to “none at all”, 6 “moderate”, and 12 “very high”. Participant ratings of delayed-onset of muscle soreness (DOMS) scale (1–10) were recorded prior to each blood sampling time point.

### Complete blood count, glucose, myoglobin

Complete blood counts (CBC) with a white blood cell (WBC) differential were performed using a Coulter Ac.T^™^ 5Diff Hematology Analyzer (Beckman Coulter, Inc., Miami, FL). Exercise-induced shifts in plasma volume were calculated using the equation of Dill and Costill [11]. Plasma glucose was measured using the YSI 2300 STAT Plus Glucose and Lactate analyzer (YSI Life Sciences, Yellow Springs, OH). All samples were analyzed in duplicate and the average of the two readings was calculated, with calibrations conducted every 5 samples or 15 minutes, whichever occurred first. Myoglobin was measured in fresh serum samples using an electrochemiluminescence immunoassay.

### Plasma cytokines

Total plasma concentrations of five inflammatory cytokines [monocyte chemoattractant protein-1 (MCP-1), IL-6, IL-8, IL-10, and IL-1 receptor antagonist (ra)] were determined using an electrochemiluminescence based solid-phase sandwich immunoassay (Meso Scale Discovery, Gaithersburg,MD, USA). All samples and provided standards were analyzed in duplicate, and the intra-assay CV ranged from 1.7% to 7.5% and the inter-assay CV ranged from 2.4 to 9.6% for all cytokines measured. Pre-and post-exercise samples were analyzed on the same assay plate to decrease inter-kit assay variability.

### Plasma 9- and 13-hydroxy-octadecadienoic acids (9+13-HODEs)

Plasma 9+13 HODEs were measured using LC-MS as previously described [12]. Chromatographic separation of 9-HODE and 13-HODE was achieved using an UPLC system (Acquity UPLC, Waters, Milford, MA) equipped with the Waters BEH C18 1.7 μm analytical column (2.1 x 100 mm). For detection of 9-HODE and 13-HODE, the UPLC system was coupled with Quattro Premier XE MS (Waters, Milford, MA) and the system was operated in electrospray ionization (ESI) negative mode.

### Global metabolomic platform materials and methods

Sample preparation, control procedures, and analysis were carried out at Metabolon Inc., as previously described [8,13]. The ultra-performance liquid chromatography-tandem mass spectrometry (UPLC-MS/MS) platform utilized a Waters Acquity UPLC with Waters UPLC BEH C18-2.1×100 mm, 1.7 μm columns and a Thermo Scientific Q-Exactive high resolution/accurate mass spectrometer interfaced with a heated electrospray ionization (HESI-II) source and Orbitrap mass analyzer operated at 35,000 mass resolution. Metabolites were identified by automated comparison of the ion features in the experimental samples to a reference library of chemical standard entries that included retention time, molecular weight (m/z), preferred adducts, and in-source fragments as well as associated MS spectra and curated by visual inspection for quality control using software developed at Metabolon. Identification of known chemical entities was based on comparison to metabolomic library entries of purified standards.

### Complex lipid panel

Lipids were extracted from 75 ul of plasma in the presence of deuterated internal standards using an automated BUME extraction according to the method of Lofgren et al. [14]. Each lipid extract was divided between two glass-lined 96-well plates (70% to Plate 1 and 30% to Plate 2), then each plate was dried under nitrogen and reconstituted in 0.25mL per sample of dichloromethane:methanol (50:50) containing 10mM ammonium acetate. Flow injection and mass spectrometry (FIA-MS) analysis was performed on a SCIEX 5500 QTRAP equipped with a SelexION Differential Mobility Separation (DMS) cell, which was operated in Multiple Reaction Monitoring (MRM) mode using both positive and negative mode electrospray in a Turbo V ion source. Plates 1 and 2 were subjected to parallel analyses, called Analysis 1 and Analysis 2, with 50 μL sample injected at a flow rate of 7 μL/min for each analysis. Analysis 1 comprised the PC, PE, LPC, LPE, PI, and SM lipid classes, while Analysis 2 comprised the CE, DAG, TAG, CER, DCER, HCER, LCER, and FFA lipid classes. Both analyses included 20 MRM cycles with 20 msec per MRM pair, a settling time of 50 msec, and a pause between mass ranges of 5 msec. Individual lipid species were quantified based on the ratio of signal intensity for target compounds to the signal intensity for an assigned internal standard of known concentration. Lipid class concentrations were calculated from the sum of all molecular species within a class, and fatty acid compositions were determined by calculating the proportion of individual fatty acids within each class.

### Anti-inflammatory in vitro assay

The human peripheral blood monocyte cell line THP-1 was obtained from ATCC (ATCC^®^ TIB-202 Livingstone, MT). THP-1 was maintained in RPMI 1640 media (Life Technologies, Grand Island, NY) supplemented with 100 IU/mL penicillin/100 μg/mL streptomycin (Thermo Fisher Scientific, Waltham, MA), 10% fetal bovine serum and 0.05 mM b-mercaptoethanol, at a density not exceeding 5 × 10^5^ cells/mL. The cells were maintained at 37°C in a humidified incubator with 5% CO_2_. Cells were subcultured into 24-well plates for the cell assay.

THP-1 monocytes were subcultured when flasks reached up to 90% confluence with a 1:5 ratio in fresh medium. Cells were seeded into 24-well plates at a concentration of 3×10^5^ cells mL^-1^ 24h prior to treatment. On the day of the experiment, medium containing the induction control (Liposacharide, LPS), positive control (Dexametasone, DEX), or plasma from the human subjects (pre- and post-exercise, 1.5 h- and 21 h-post-exercise) were added to a set of 3 wells per sample and incubated for 6 h at 37 °C with 5% CO_2_. Three replicates were made for both the treatments and the controls. At the end of the treatment period, cells were harvested in TRIzol reagent (Life Technologies) for subsequent cellular RNA extraction. Cells were seeded in a 96-well plate for the cell viability assay. Cell viability was measured by MTX assay in triplicate and quantified spectrophotometrically using a Synergy H1 microplater reader (BioTek, Winooski, VT).

### RNA extraction, cDNA synthesis, and qPCR

Total RNA was isolated from monocytes using Trizol reagent (Life Technologies) following the manufacturer’s instructions. RNA was quantified spectrophotometrically using the SynergyH1/Take3 (BioTek). The cDNAs were synthesized with 2.0 μg of RNA using commercially available high-capacity cDNA Reverse Transcription Kit (Life Technologies), following the manufacturers’ protocols on an ABI Gene AMP 9700 (Life Technologies). The resulting cDNA was amplified by real-time quantitative PCR using Power SYBR green PCR Master Mix (Life Technologies). To avoid interference due to genomic DNA contamination, only intron-overlapping primers were selected. Quantitative PCR was performed in duplicate using the following gene-specific primers as follows: GAPDH, forward primer 5’-ATG GGG AAG GTG AAG GTC G-3’, reverse primer 5’-TAA AAG CAG CCC TGG TGA CC -3’; Cox-2, forward primer 5’-GCT GGA ACA TGG AAT TAC CCA-3’, reverse primer 5’-CTT TCT GTA CTG CGG GTG GAA-3’. Quantitative PCR (qPCR) amplifications were performed on an ABI7500 Fast real time PCR (Life Technologies) using 1 cycle at 50 °C for 2 min and 1 cycle of 95 °C for 10min, followed by 40 cycles of 15s at 95°C and 1 min at 60 °C. Samples were subjected to a melting curve analysis to confirm the amplification specificity. The dissociation curve was completed with 1 cycle of 1 min at 95 °C, 30 s at 55 °C, and 30 s at 95 °C. mRNA expression was analyzed using the ΔΔCT method and normalized with respect to the expression of the GAPDH housekeeping genes using 7500 Fast System SDS software v1.3.0 (Life Technologies). A value of <1.0 indicates transcriptional down-regulation (inhibition of gene expression) compared with control cells, which shows maximum genetic induction (1.0). Therefore, lower values indicate greater anti-inflammatory activity. Values of >1.0 implies overexpression of the particular gene. Amplification of specific transcripts was further confirmed by obtaining melting curve profiles.

### Measurement of cellular bioenergetics and oxidative burst

The human peripheral blood monocyte cell line THP-1 (5 × 10^4^ cells per well) were seeded in 24 well XF assay plates and subjected to real-time measurements of oxygen consumption rate (OCR) and extracellular acidification rate (ECAR) using the XF 24 Extracellular Flux Analyzer (Seahorse Biosciences, North Billerica, MA). Cells were then added to 500 μl of XF assay medium (DMEM without NaHCO_3_, 10 mM glucose, 2 mM pyruvate, pH 7.4), and equilibrated at non-CO_2_ incubator and 37°C for 1 h. Following a triplicate 6-hour treatment with plasma samples obtained immediately post-exercise (50 μL/mL of a pooled sample obtained from the athletes), 21 measurements were performed over 460 minutes under basal, LPS-stimulated conditions, and the addition of mitochondrial inhibitors, with the first three discarded. OCR and ECAR were automatically recorded by Seahorse XF24 software v1.8. Basal and LPS stimulated (10 ng/mL) OCR and ECAR rates were determined by averaging the data points obtained in those phases. Following LPS stimulation, the mitochondrial complex inhibitors were injected sequentially in the following order: oligomycin (1 μM), FCCP (0.75 μM), antimycin A (1 μM each), and the readings were taken after each inhibitor [15]. Spare respiratory capacity was calculated with this formula: [(maximal respiration)/(basal respiration)x100] (https://www.agilent.com/en/promotions/seahorse-xf-technology).

### Statistical analysis

Data are presented as mean±standard error (SE). Male and female subject characteristics, and food record and performance data across conditions were compared using paired t-tests. Biomarker data were analyzed using a 4 (condition) x 8 (time) repeated-measures ANOVA, within-participants design, with changes over time within conditions contrasted between conditions using paired t-tests. Data were checked for normality of the residuals using Q-Q plots. For the metabolomics data, raw area counts for each metabolite in each sample were normalized to correct for variation resulting from instrument inter-day tuning differences. Median values for each run-day were set to 1.0. Missing values were imputed with the observed minimum after normalization. Following log transformation of the normalized data, analysis by two-way ANOVA with repeated measures with post-test contrasts was performed and statistical significance was set at q<0.05. A false discovery rate estimate (“q-value”) was calculated to adjust for multiple comparisons.^26^ Fold changes across time points were calculated using group averages of the median scaled intensity values. These statistical procedures were performed using SAS (SAS Institute, Cary, NC).

Orthogonal Partial Least Square Discriminant Analysis (OPLS-DA) in SIMCA (Version 14.1, Umetrics, Umeå, Sweden) was used to detect metabolites that best distinguished the time points and/or the trials. The default 7-round cross-validation in SIMCA was used to compute the diagnostic Q^2^Y value which is a measure of model prediction ability. Permutation based validation was used to prevent overfitting. During permutation based validation, the prediction ability of the model was compared with the prediction ability of each model built using each of the 999 permutated data sets. The separation observed in OPLS-DA was considered statistically significant if its prediction ability was found to be better than 95% of the models built using the permutated data sets. Variable Influence on Projection (VIP) score was calculated for each metabolite based on its contribution to the model. Metabolites with VIP>2.5 were considered important metabolites in explaining the separation. For comparison between time points in OPLS-DA, the normalized metabolomics data was used. For comparison between trials in OPLS-DA, ratios between immediate post-exercise and pre-exercise were calculated for each subject and used as input data in the analysis.

Statistical analyses for human peripheral blood monocyte cell line THP-1 (anti-inflammatory in vitro assay and bioenergetics assay) were performed using Prism 6.0 (GraphPad Software, San Diego, CA). Data were analyzed by two-way ANOVA with treatment as a factor. Post hoc analyses of differences between individual experimental groups were made using the Dunnett’s multiple comparison tests. Significant differences were accepted when the p-value was <0.05.

## Results

The analysis included 20 male cyclists (14 males, 6 females) who successfully adhered to all aspects of the study design (see Table 1). The study participant number (N = 20) provided 84% power to detect a difference with an effect size 0.7 at alpha 0.05 using two sided paired t-tests. The male and female cyclists did not differ in training volumes, body composition, and VO_2max_. Three-day food records collected before each of the four 75-km cycling time trials revealed no significant differences in energy, carbohydrate, micronutrient intake, or flavonoid intake (data not shown). Across all four trials, energy intake averaged 2093±51.4 kcal/day (8.76±0.2 MJ/day), with carbohydrate representing 45.7±0.8% of total energy, and flavonoid intake averaging 85.7±21.4 mg/day.

**Table 1:** Participant characteristics (mean±SE).

Variable	Males (N = 14)	Females (N = 6)	P-value
Age (years)	37.1±2.5	43.7±2.2	0.126
Height (m)	1.81±1.6	1.63±4.5	<0.001
Weight (kg)	81.0±2.8	62.7±2.1	<0.001
Body fat (%)	19.5±1.3	18.8±1.9	0.763
Watts_max_	277±8.9	238±14.1	0.027
VO_2max_ (ml·kg·^-1^min^-1^)	47.0±1.5	46.5±2.8	0.861
HR_max_ (beats/min)	171±1.9	163±3.3	0.023
Training (km/wk)	118±13.6	136±24.1	0.520

**Fig 2:**
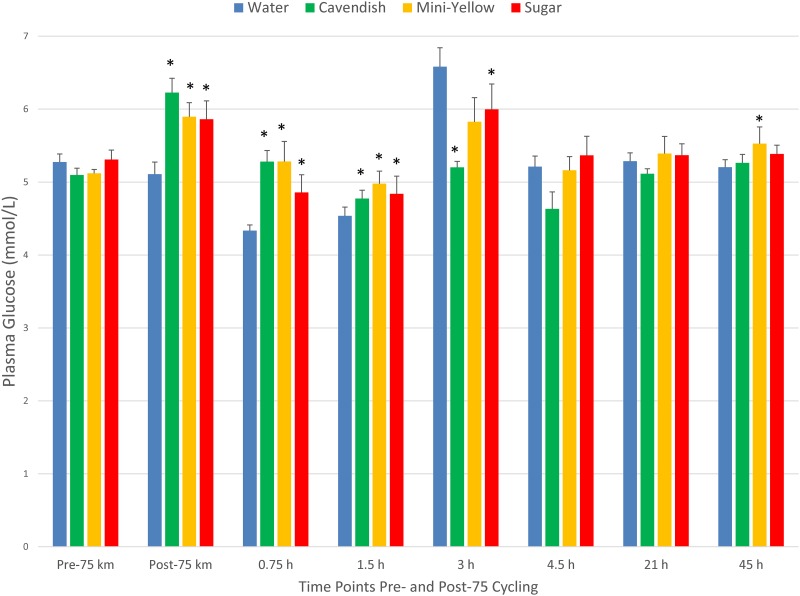
Changes in plasma glucose during 45-h recovery from 75-km cycling across four treatments (water only, Cavendish and mini-yellow bananas, and 6% sugar beverage) in 20 cyclists. Interaction effect, P<0.001. * P<0.017 compared to the change from pre-exercise in the water condition.

Table 2 summarizes performance data for the four 75-km cycling bouts. Performance times, absolute oxygen consumption (L/min), heart rates, the rating of perceived exertion (RPE), and plasma volume shift did not differ during the two banana and sugar beverage trials compared to the water condition. Absolute and relative power (watts) was slightly higher for the Cavendish banana compared to water condition. The respiratory exchange ratios (RER) were significantly elevated in both the mini-yellow banana and sugar beverage trials relative to the water trial. Plasma glucose was significantly elevated during the first 1.5 h post-exercise in the two banana and sugar beverage trials compared to the water trial, with a significant rebound in plasma glucose in the water condition following lunch (consumed after the 1.5 h post-exercise blood collection) (interaction effect, P<0.001) (Fig 2). Participants reported more fullness and bloating, and reduced muscle cramping, following the two banana and sugar beverage trials compared to the water trial (Table 2). No trial differences were found for symptoms of diarrhea or nausea/vomiting (data not shown). Delayed onset of muscle soreness (DOMS) ratings increased post-exercise (time effect, P<0.001), with lower levels found for the mini-yellow banana and sugar beverage trials at the 1.5 h post-exercise time point (interaction effect, P = 0.014) (data not shown).

**Table 2:** Metabolic, performance, and symptom data during the 75-km cycling trials under water, Cavendish banana, mini-yellow banana, and sugar beverage conditions in trained cyclists (N = 20) (mean±SE).

Variable	Water	Cavendish Banana	Mini-Yellow Banana	Sugar Beverage
Time (minutes)	178±3.7	180±4.8	184±4.4	176±4.5
VO_2_ (L/min)	2.45±0.93	2.53±0.89	2.41±0.85	2.62±1.06
VO_2_ (%VO_2max_)	70.5±2.1	73.8±2.3	69.5±2.3	75.0±2.3*
Watts	195±9.3	208±8.2*	204±9.3	204±9.3
% Watts_max_	57.5±2.0	61.5±1.5*	60.1±2.0	60.1±2.0
HR (beats/min)	143±2.8	142±2.6	139±3.1	140±3.4
RPE	13.8±0.3	13.8±0.3	13.2±0.2	13.6±0.3
RER at 55 km	0.81±0.01	0.84±0.01	0.86±0.01*	0.86±0.01*
Plasma volume shift (%)	-11.1±1.5	-12.2±1.2	-13.0±1.5	-8.1±1.0
Post-exercise symptom shift				
Feeling full	-1.1±0.5	3.0±0.6*	3.2±0.5*	0.3±0.6*
Bloating	-0.4±0.4	1.9±0.5*	2.9±0.7*	0.8±0.4*
Energy level	-3.6±0.7	-1.9±0.7*	-2.4±0.5	-2.4±0.4
Muscle cramping	3.7±0.6	1.8±0.5*	2.0±0.4*	2.3±0.4*

* P<0.017 compared to the water condition.

**Fig 3:**
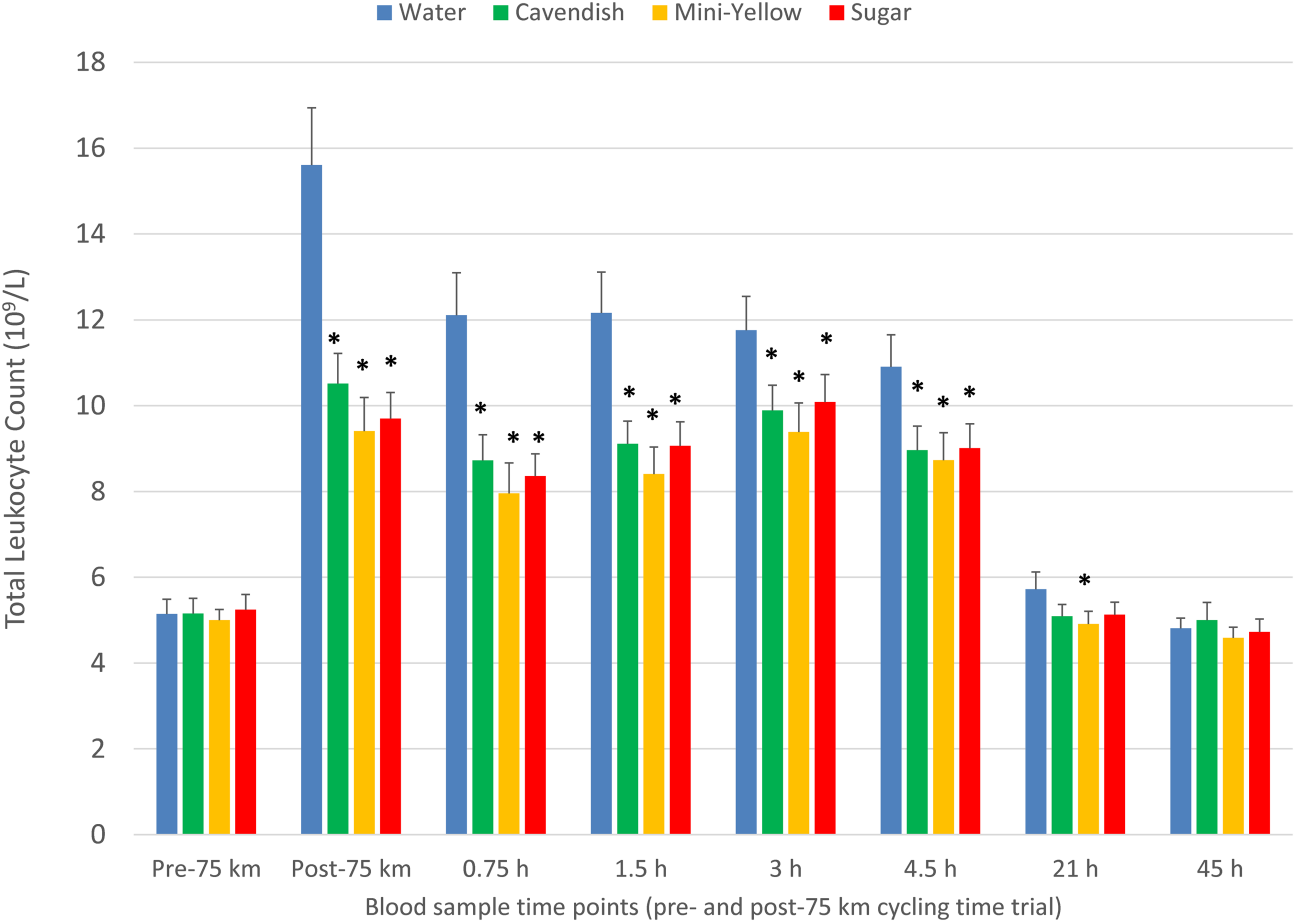
Changes in total blood leukocytes during 45-h recovery from 75-km cycling across four treatments (water only, Cavendish and mini-yellow bananas, and 6% sugar beverage) in 20 cyclists. Interaction effect, P<0.001. * P<0.017 compared to the change from pre-exercise in the water condition.

**Fig 4:**
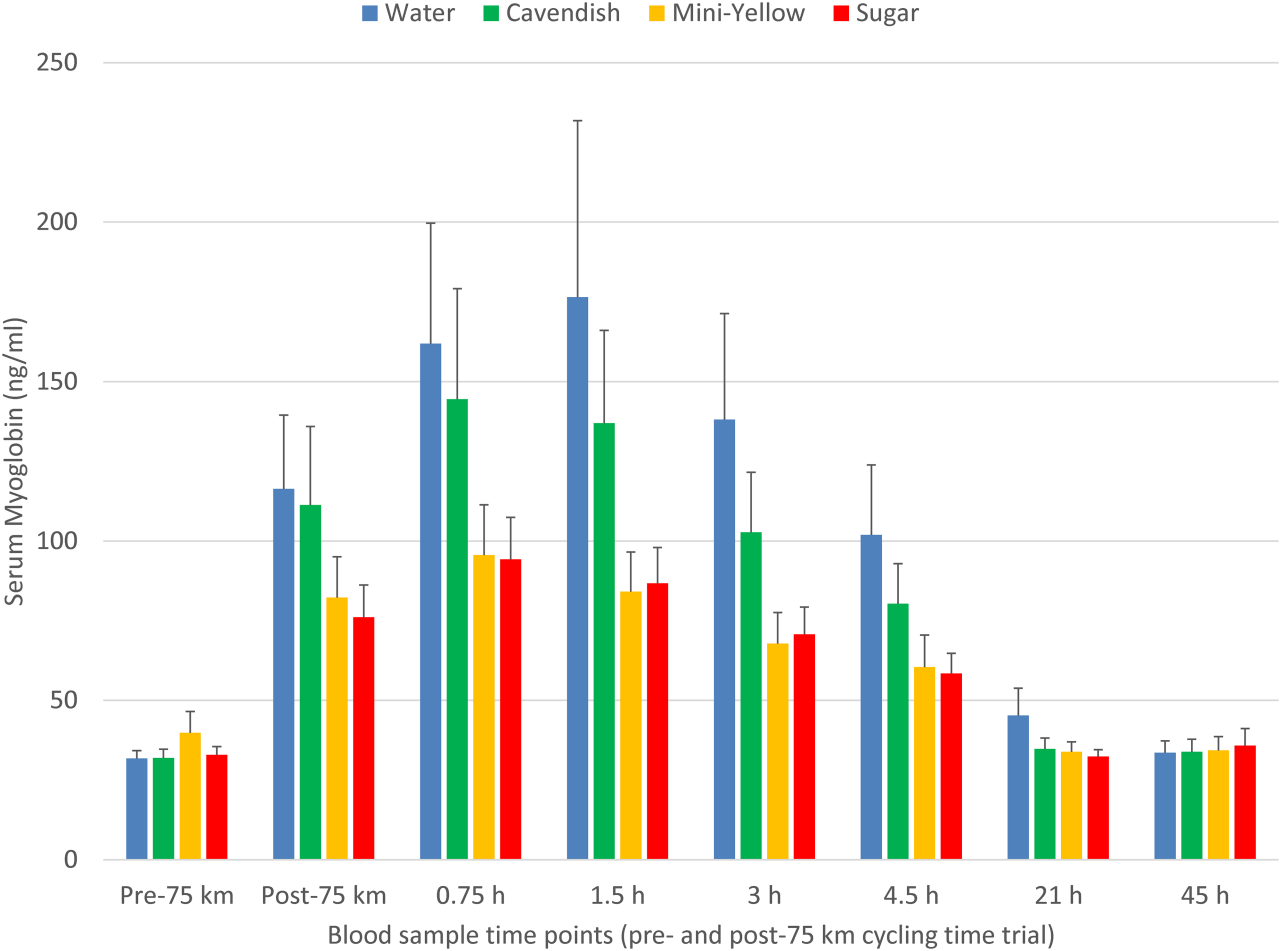
Changes in serum myoglobin during 45-h recovery from 75-km cycling across four treatments (water only, Cavendish and mini-yellow bananas, and 6% sugar beverage) in 20 cyclists. Interaction effect, P = 0.002.

**Fig 5:**
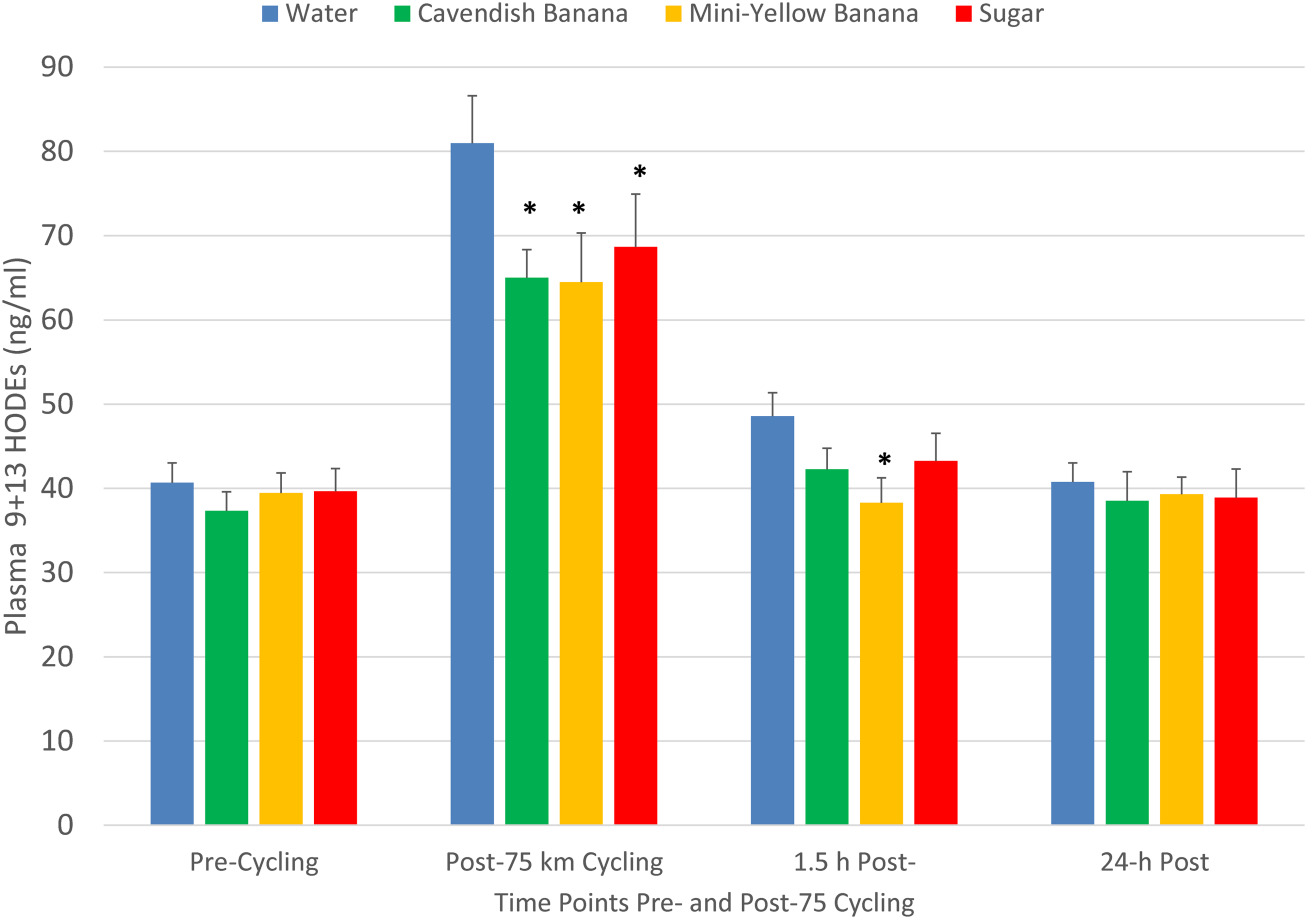
Changes in plasma 9+13 HODEs during 45-h recovery from 75-km cycling across four treatments (water only, Cavendish and mini-yellow bananas, and 6% sugar beverage) in 20 cyclists. Interaction effect, P<0.001. * P<0.017 compared to the change from pre-exercise in the water condition.

**Fig 6:**
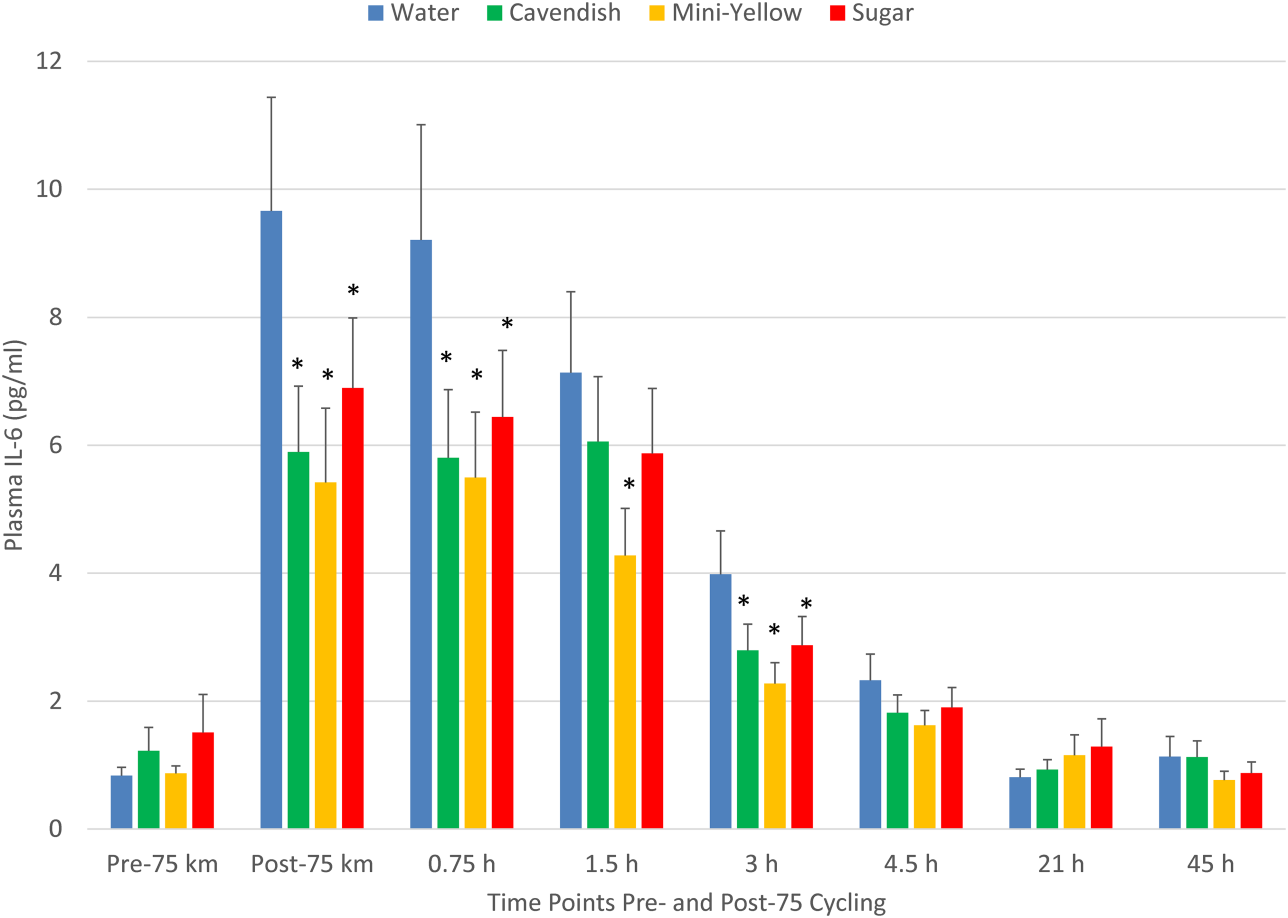
Changes in plasma IL-6 during 45-h recovery from 75-km cycling across four treatments (water only, Cavendish and mini-yellow bananas, and 6% sugar beverage) in 20 cyclists. Interaction effect, P<0.001. * P<0.017 compared to the change from pre-exercise in the water condition.

The pattern of increase in post-exercise total blood leukocyte counts was significantly different between trials (interaction effect, P<0.001), with lower levels during the first 4.5 h recovery for the two banana and sugar beverage trials compared to water (Fig 3). A similar trial contrast was found for serum myoglobin (interaction effect, P = 0.009), but time point contrasts were non-significant due to substantial variation (Fig 4). The increase in plasma 9+13 HODEs immediately post-exercise was attenuated for the two banana and sugar beverage trials compared to the water trial (interaction effect, P<0.001) (Fig 5). Interaction effects were significant for plasma IL-6, IL-8, IL-10, IL-1ra, and MCP-1 (Fig 6 and Table 3), with substantial post-exercise reductions at selected time points for the two banana and sugar beverage versus water trials.

**Table 3:** Comparison between water, Cavendish banana, mini-yellow banana, and sugar beverage trials for cytokine inflammation biomarkers in trained cyclists (N = 20) before and during recovery from cycling 75-km (mean±SE).

Variable	Water	Cavendish banana	Mini-Yellow Banana	Sugar Beverage	P-values: Interaction
**IL-8**					
Pre-exercise	4.65±0.5	4.91±0.7	5.03±0.5	4.46±0.4	0.020
Immediate post-exercise	12.5±1.7	9.94±1.3*	10.2±1.2	9.48±1.3*	
0.75 h post-exercise	11.7±1.4	10.4±1.2	10.1±1.0	9.43±1.1	
1.5 h post-exercise	10.3±1.6	8.23±1.1	8.42±0.8	7.96±0.9	
3.0 h post-exercise	6.76±0.9	5.90±0.8	6.26±0.9	6.14±1.1	
4.5 h post-exercise	6.48±1.4	5.97±0.8	5.84±0.6	5.20±0.7	
21 h post-exercise	4.75±0.5	4.51±0.4	5.01±0.5	4.78±0.5	
45 h post-exercise	4.08±0.4	4.74±0.6	5.20±0.6	4.67±0.5	
**IL-10 (pg/ml)**					
Pre-exercise	4.65±0.5	4.91±0.7	5.03±0.5	4.46±0.4	0.003
Immediate post-exercise	12.5±1.7	9.94±1.3*	10.2±1.2*	9.48±1.3*	
0.75 h post-exercise	11.7±1.4	10.4±1.2*	10.1±1.0*	9.43±1.1*	
1.5 h post-exercise	10.3±1.6	8.23±1.1*	8.42±0.8	7.96±0.9	
3.0 h post-exercise	6.76±0.9	5.90±0.8	6.26±0.9	6.14±1.1	
4.5 h post-exercise	6.48±1.4	5.97±0.8	5.84±0.6	5.20±0.7	
21 h post-exercise	4.75±0.5	4.51±0.4	5.01±0.5	4.78±0.5	
45 h post-exercise	4.08±0.4	4.74±0.6	5.20±0.6	4.67±0.5	
**IL-1ra (pg/ml)**					
Pre-exercise	105±9.8	114±14.6	118±12.4	114±11.4	0.040
Immediate post-exercise	681±309	263±82.7	184±25.4	185±24.1*	
0.75 h post-exercise	1001±334	211±27.9	224±47.0	272±77.8	
1.5 h post-exercise	1222±349	218±34.8*	259±67.5*	303±84.9*	
3.0 h post-exercise	1214±352	203±19.3*	283±72.7*	267±52.1*	
4.5 h post-exercise	729±307	184±30.6	160±22.2	191±29.4	
21 h post-exercise	126±13.5	103±12.0	114±14.4	107±10.8	
45 h post-exercise	103±9.1	115±13.9	104±10.5	106±10.4	
**MCP-1**					
Pre-exercise	245±18.9	230±21.5	252±17.2	235±15.8	0.010
Immediate post-exercise	363±21.7	306±30.0*	315±19.7	315±21.5	
0.75 h post-exercise	344±21.1	305±28.1	314±18.8	300±21.2	
1.5 h post-exercise	319±25.0	287±27.0	290±19.2	273±21.4	
3.0 h post-exercise	270±28.4	247±27.4	248±19.0	252±21.2	
4.5 h post-exercise	226±32.0	228±31.8	240±22.7	224±22.8	
21 h post-exercise	187±26.9	196±26.6	199±21.3	200±21.9	
45 h post-exercise	206±24.8	210±16.6	223±19.6	217±19.1	

IL = interleukin; IL-1ra = interleukin 1 receptor antagonist; MCP-1 = monocyte chemoattractant protein 1.

* P<0.017 compared to the change from pre-exercise in the water condition.

**Fig 7:**
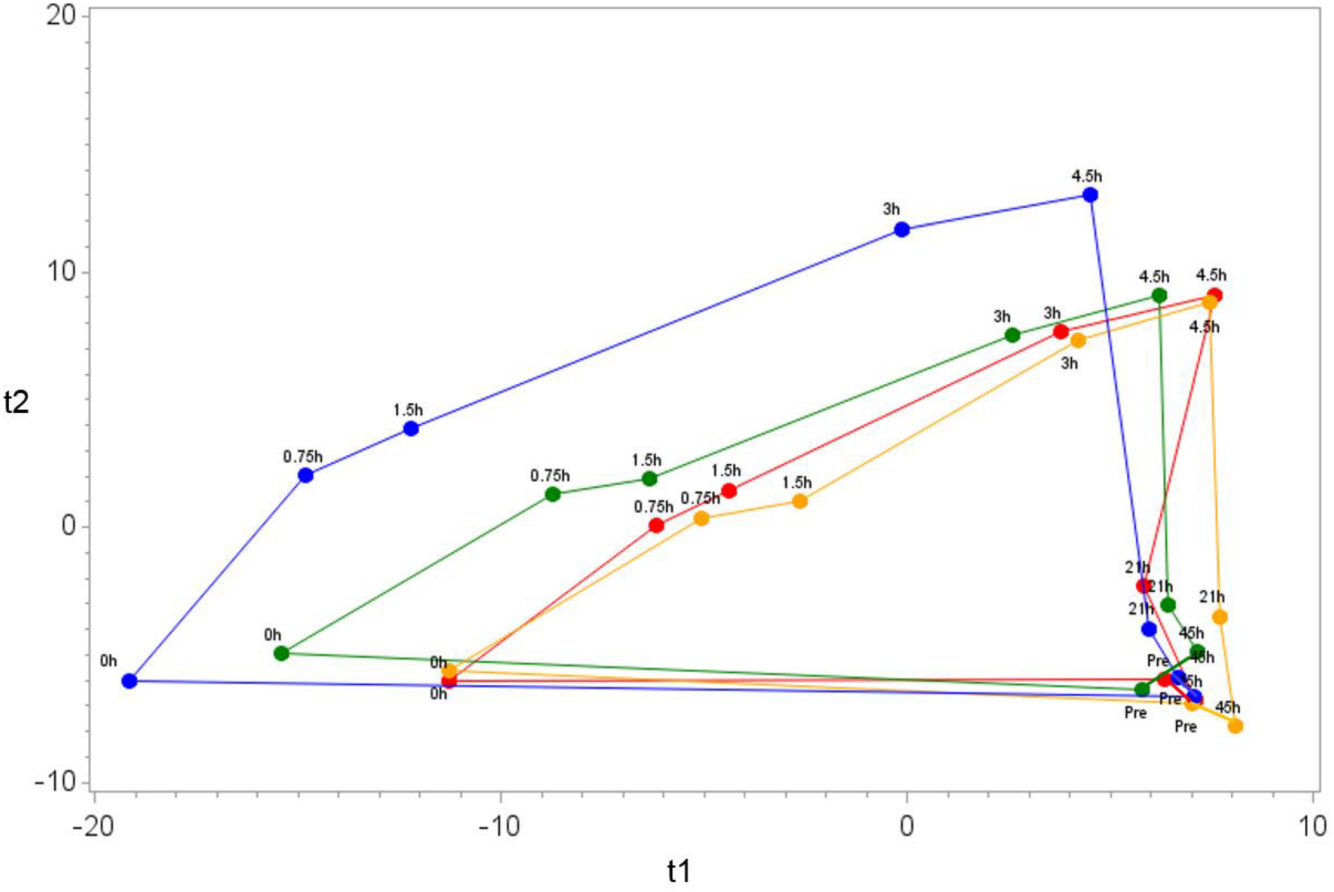
OPLS-DA and the overall pattern in the data. The centroid was calculated for the samples in each treatment and time point combination. Water trial is in blue; 6% sugar beverage trial is in red; Cavendish bananas trial is in green; Mini-yellow banana trial is in yellow. This analysis passed permutation based validation, which indicates that the observed pattern did not occur by chance. However, the low Q2Y score (0.049) indicates poor prediction ability, especially for the time points that have similar metabolomics profiles, such as pre-exercise, 21-h and 45-h post-exercise. It is not a concern in this analysis, because complete separations of all of the treatment and time point combinations were not expected. The goal of this analysis was to examine the overall pattern in the data.

Metabolomics analysis revealed 1,605 biochemicals of known identity from the global metabolomics and complex lipid platforms (S1 Table). Fig 7 depicts the centroid plot using OPLS-DA for all trials, sampling time points, and biochemicals. The length of the full loop for the water trial (86.8±4.0 arbitrary units) was significantly greater (i.e., indicating a greater and more sustained metabolic perturbation) than for the Cavendish banana (70.4±3.9, P = 0.006), mini-yellow banana (68.3±4.0, P = 0.002), and sugar beverage trials (68.1±4.2, P = 0.002).

A total of 109 metabolites increased more than 2.0-fold and 71 metabolites decreased by more than 0.5-fold immediately after the 75-km cycling time trial during the water-only trial (q≤0.05) (S2 Table). The majority of the 109 metabolites increasing immediately post-exercise were from the lipid super pathway, with 65% of the 71 metabolites decreasing identified as triacylglycerol esters and 14% as primary and secondary bile acids.

The influence of ingesting carbohydrate during exercise on metabolite shifts relative to water intake is summarized in Table 4 and S3 Table. The fold change in metabolites immediately post-exercise was ranked by VIP when comparing sugar beverage and water intake, with the top 30 metabolites listed in Table 4 (VIP>2.5, q<0.05). Post-exercise fold changes are also included for the two types of bananas for comparison. All three carbohydrate sources were associated with significant increases for fructose, sucrose, and glucose, and significant decreases for multiple metabolites from the lipid super-pathway, the amino acid isoleucine, and the ratio 2-hydroxybutyrate/2-hydroxyisobutyrate from the glutathione sub-pathway. Plasma cortisol levels were significantly reduced 19 to 39% during the first 1.5 h of recovery from exercise with carbohydrate ingestion (all three sources) compared to water (S3 Table).

**Table 4:** Fold change in metabolites immediately post-exercise (ranked by VIP>2.5, with q<0.05) by carbohydrate intake from the sugar beverage (sugar) compared to water. Post-exercise fold changes also included for the two types of bananas for comparison (CAV = Cavendish, MiniY = mini-yellow) compared to water. Dark red (increase) and green (decrease) cells represent q<0.05 contrasts from pre-exercise levels. (FFA = free fatty acid; FA = fatty acid). See also S3 Table.

Biochemical Name	SUPER PATHWAY	SUB PATHWAY	VIP rank	Sugar vs Water	CAV vs Water	MiniY vs Water
hexanoylglutamine	Lipid	FA Metabolism (Acyl Glutamine)	3.46	**0.37**	**0.49**	**0.23**
3-hydroxybutyrate (BHBA)	Lipid	Ketone Bodies	3.28	**0.28**	**0.39**	**0.23**
2-hydroxybutyrate/2-hydroxyisobutyrate	Amino Acid	Glutathione Metabolism	3.20	**0.59**	**0.66**	**0.53**
3-aminoisobutyrate	Nucleotide	Pyrimidine Metabolism, Thymine containing	3.17	**0.71**	0.92	**0.76**
fructose	Carbohydrate	Fructose, Mannose and Galactose Metabolism	3.13	**6.79**	**5.14**	**5.86**
dodecanedioate	Lipid	FA, Dicarboxylate	3.10	**0.27**	**0.37**	**0.25**
leucine	Amino Acid	Leucine, Isoleucine and Valine Metabolism	3.00	**0.81**	0.89	0.89
5-bromotryptophan	Amino Acid	Tryptophan Metabolism	2.96	**1.39**	**1.44**	**1.67**
erythronate	Carbohydrate	Aminosugar Metabolism	2.94	**1.80**	**1.59**	**1.65**
isoleucine	Amino Acid	Leucine, Isoleucine and Valine Metabolism	2.92	**0.83**	**0.84**	**0.87**
FFA(17:0)	FFA	FFA	2.92	**0.82**	**0.86**	**0.76**
oleoylcarnitine (C18:1)	Lipid	FA Metabolism(Acyl Carnitine)	2.92	**0.64**	**0.79**	**0.58**
tetradecanedioate	Lipid	FA, Dicarboxylate	2.91	**0.32**	**0.39**	**0.25**
16-hydroxypalmitate	Lipid	FA, Monohydroxy	2.84	**0.61**	**0.64**	**0.48**
suberate (octanedioate)	Lipid	FA, Dicarboxylate	2.83	**0.56**	**0.72**	**0.51**
hexadecanedioate	Lipid	FA, Dicarboxylate	2.81	**0.38**	**0.45**	**0.26**
glycerol	Lipid	Glycerolipid Metabolism	2.80	**0.68**	0.76	**0.56**
cinnamoylglycine	Xenobiotics	Food Component/Plant	2.77	**0.52**	**0.35**	**0.45**
3-hydroxyoctanoate	Lipid	FA, Monohydroxy	2.76	**0.62**	**0.80**	**0.59**
Total FFA	Complex Lipids	FFA	2.76	**0.80**	**0.85**	**0.72**
3-hydroxyhexanoate	Lipid	FA, Monohydroxy	2.75	**0.70**	**1.40**	**1.40**
gamma-glutamylleucine	Peptide	Gamma-glutamyl Amino Acid	2.71	**0.72**	0.93	0.85
phenylalanyltryptophan	Peptide	Dipeptide	2.70	**0.76**	0.86	**0.73**
3-hydroxysebacate	Lipid	FA, Monohydroxy	2.62	**0.33**	0.52	**0.25**
sucrose	Carbohydrate	Disaccharides and Oligosaccharides	2.61	**15.41**	**8.75**	**11.46**
glucose	Carbohydrate	Glycolysis, Gluconeogenesis, and Pyruvate Metabolism	2.58	**1.29**	**1.30**	**1.29**
3-hydroxyisobutyrate	Amino Acid	Leucine, Isoleucine and Valine Metabolism	2.58	**0.57**	0.85	**0.59**
FFA(14:0)	FFA	FFA	2.54	**0.78**	0.84	**0.68**
sebacate (decanedioate)	Lipid	FA, Dicarboxylate	2.53	**0.30**	**0.36**	**0.22**
FFA(16:0)	FFA	FFA	2.52	**0.84**	0.88	**0.77**

Table 5 and S4 Table provides fold changes for metabolites most effected by banana ingestion. The VIP ranking is based on metabolite shifts immediately post-exercise for the intake of the cavendish banana versus water, with comparisons relative to the carbohydrate beverage for both bananas. Both types of bananas were associated with significant fold changes for several metabolites from the amino acid super-pathway including 2-oxoarginine, pipecolate, argininate, dopamine 3-O-sulfate, asparagine, tyramine O-sulfate, 3-methoxytyramine, 5-hydroxyindoleacetate, and S-methylmethionine. Other unique metabolites associated with banana intake were increases in 2-isopropylmalate and 2,3-dihydroxyisovalerate (food plant component), pyridoxate (vitamin B6 metabolism), and trigonelline (nicotinate metabolism).

**Table 5:** Fold change in metabolites immediately post-exercise (ranked by VIP>3.0) by intake of Cavendish (CAV) banana compared to water. Post-exercise fold changes also included for mini-yellow (MiniY) versus water, and CAV and MiniY versus the sugar beverage. Dark red (increase) and green (decrease) cells represent q<0.05 contrasts from pre-exercise levels. (FFA = free fatty acid; FA = fatty acid). See also S4 Table.

BIOCHEMICAL	SUPER PATHWAY	SUB PATHWAY	VIP rank	CAV vs Water	CAV vs Sugar	MiniY vs Water	MiniY vs Sugar
2-oxoarginine	Amino Acid	Urea cycle; Arginine and Proline Metabolism	3.73	**7.74**	**7.78**	**10.1**	**10.1**
pipecolate	Amino Acid	Lysine Metabolism	3.73	**1.99**	**1.83**	**2.52**	**2.32**
argininate	Amino Acid	Urea cycle; Arginine and Proline Metabolism	3.71	**1.84**	**1.66**	**2.14**	**1.93**
dopamine 3-O-sulfate	Amino Acid	Tyrosine Metabolism	3.67	**21.1**	**20.0**	**24.5**	**23.1**
fructose	Carbohydrate	Fructose, Mannose and Galactose Metabolism	3.51	**5.14**	**0.76**	**5.86**	0.86
2-isopropylmalate	Xenobiotics	Food Component/Plant	3.43	**245**	**166**	**58.4**	**39.6**
asparagine	Amino Acid	Alanine and Aspartate Metabolism	3.36	**1.21**	**1.11**	**1.19**	**1.08**
tyramine O-sulfate	Amino Acid	Tyrosine Metabolism	3.36	**5.88**	**7.41**	**10.6**	**13.3**
5-bromotryptophan	Amino Acid	Tryptophan Metabolism	3.35	**1.44**	1.03	**1.67**	**1.20**
3-hydroxybutyrate (BHBA)	Lipid	Ketone Bodies	3.32	**0.39**	1.42	**0.23**	0.82
3-methoxytyramine sulfate	Amino Acid	Tyrosine Metabolism	3.32	**2.73**	**3.60**	**2.54**	**3.35**
erythronate	Carbohydrate	Aminosugar Metabolism	3.23	**1.59**	**0.88**	**1.65**	**0.92**
cinnamoylglycine	Xenobiotics	Food Component/Plant	3.19	**0.35**	**0.68**	**0.45**	0.87
16-hydroxypalmitate	Lipid	Fatty Acid, Monohydroxy	3.15	**0.64**	1.06	**0.48**	**0.78**
2,3-dihydroxyisovalerate	Xenobiotics	Food Component/Plant	3.15	**40.5**	**36.7**	**3.80**	**3.44**
isoleucine	Amino Acid	Leucine, Isoleucine and Valine Metabolism	3.12	**0.84**	1.02	**0.87**	1.05
histidine	Amino Acid	Histidine Metabolism	3.12	**1.17**	**1.07**	**1.25**	**1.14**
5-hydroxyindoleacetate	Amino Acid	Tryptophan Metabolism	3.11	**18.0**	**17.5**	**66.3**	**64.3**
trigonelline (N’-methylnicotinate)	Cofactors and Vitamins	Nicotinate and Nicotinamide Metabolism	3.09	**2.70**	**2.75**	**2.73**	**2.78**
serine	Amino Acid	Glycine, Serine and Threonine Metabolism	3.08	**1.11**	**1.07**	**1.17**	**1.13**
kynurenine	Amino Acid	Tryptophan Metabolism	3.08	**1.21**	**1.09**	**1.29**	**1.16**
pyridoxate	Cofactors and Vitamins	Vitamin B6 Metabolism	3.07	**2.50**	**2.79**	**1.72**	**1.92**
gamma-glutamylalanine	Peptide	Gamma-glutamyl Amino Acid	3.05	**1.34**	**1.17**	**1.49**	**1.31**
S-methylmethionine	Amino Acid	Methionine, Cysteine, SAM and Taurine Metabolism	3.05	**45.8**	**35.8**	**6.98**	**5.46**
dodecanedioate	Lipid	Fatty Acid, Dicarboxylate	3.04	**0.37**	**1.37**	**0.25**	0.92

**Fig 8:**
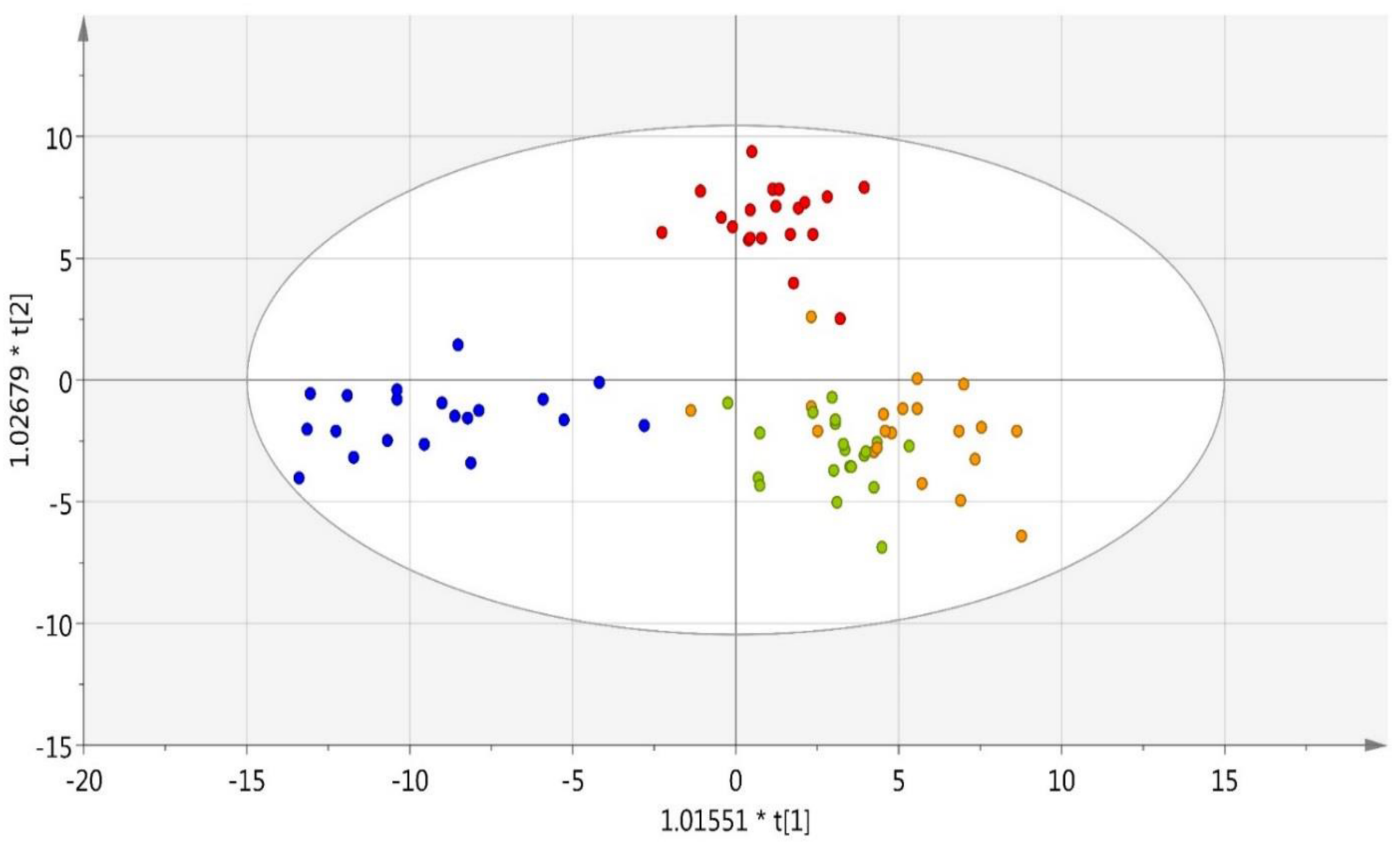
OPLS-DA for the separation between water trial (blue), 6% sugar beverage trial (red), Cavendish bananas trial (green), and mini-yellow banana trial (yellow). R2Y = 0.848, Q2Y = 0.409. This analysis passed permutation based validation. The ratio between immediately post-exercise and pre-exercise for each metabolite in each subject was calculated and used as input data for this analysis. Metabolites important for the separation are listed in S5 Table.

**Fig 9:**
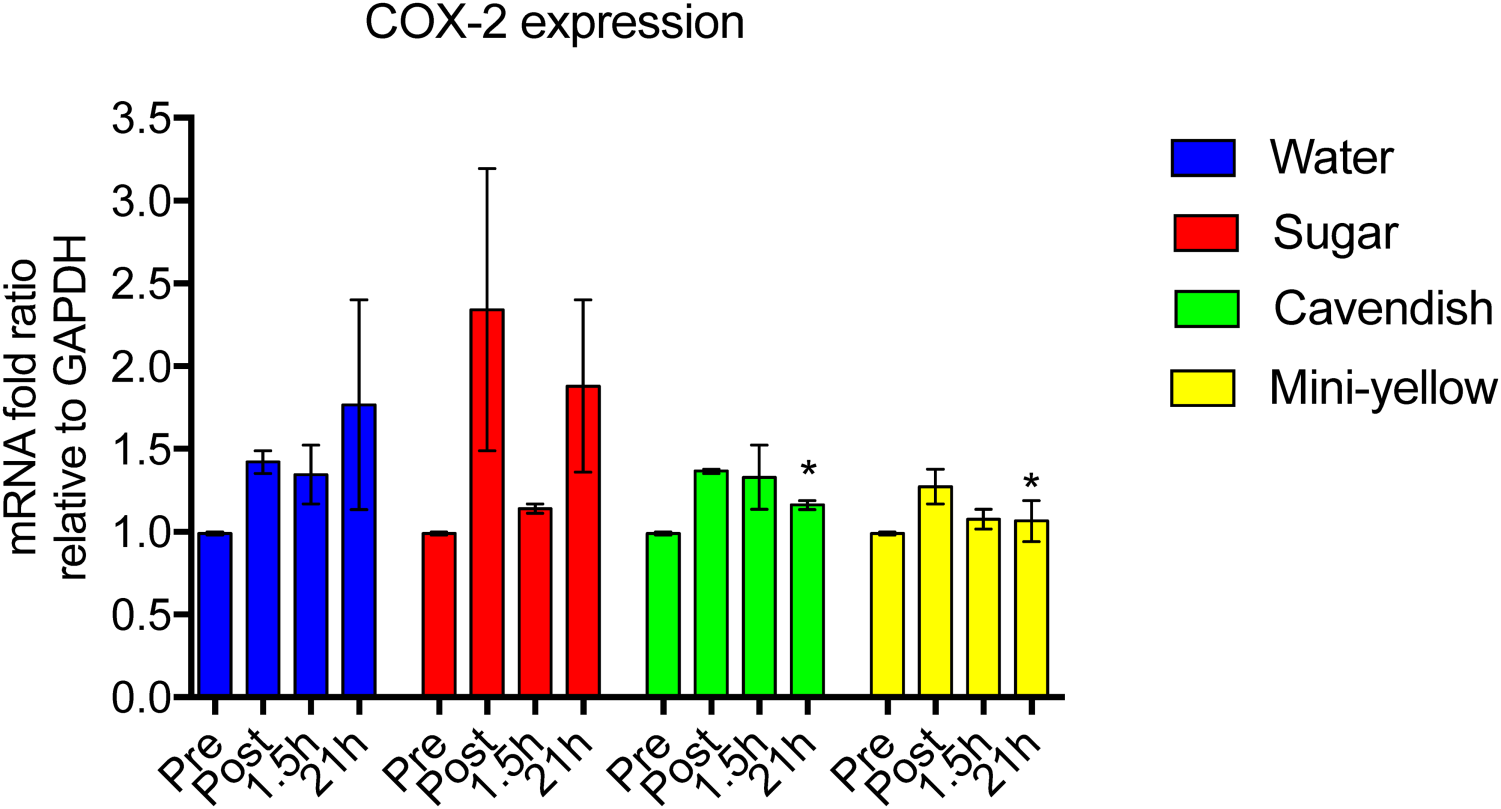
COX-2 mRNA expression in THP-1 monocytes cultured for six hours in plasma obtained from study participants pre- and immediately-post-exercise, and 1.5 h- and 21-h post-exercise across four treatments (water only, Cavendish and mini-yellow bananas, and 6% sugar beverage). Data are mean ± SE expressed as mRNA fold change relative to GAPDH. * P≤ 0.05 compared to the change from the water-only trial.

Immediate post-exercise change ratios were calculated for each metabolite in each subject, and used as input data for OPLS-DA analysis. Fig 8 depicts the significant separation between the four trials (R2Y = 0.848, Q2Y = 0.409). This analysis passed permutation based validation. Metabolites important for the separation are listed in S5 Table. These data indicate that metabolome shifts were distinctly different with the two banana trials compared to both water alone and the sugar beverage, and additional assays were conducted to determine if the plasma samples collected post-exercise differed in two inflammation-related outcomes. Fig 9 depicts COX-2 mRNA expression in THP-1 monocytes cultured for six hours in plasma obtained from study participants pre- and immediately-post-exercise, and 1.5 h- and 21-h post-exercise across the four trials. Relative to the water only trial, COX-2 mRNA expression in THP-1 monocytes was significantly decreased 21-h post-exercise in both of the banana trials.

**Fig 10:**
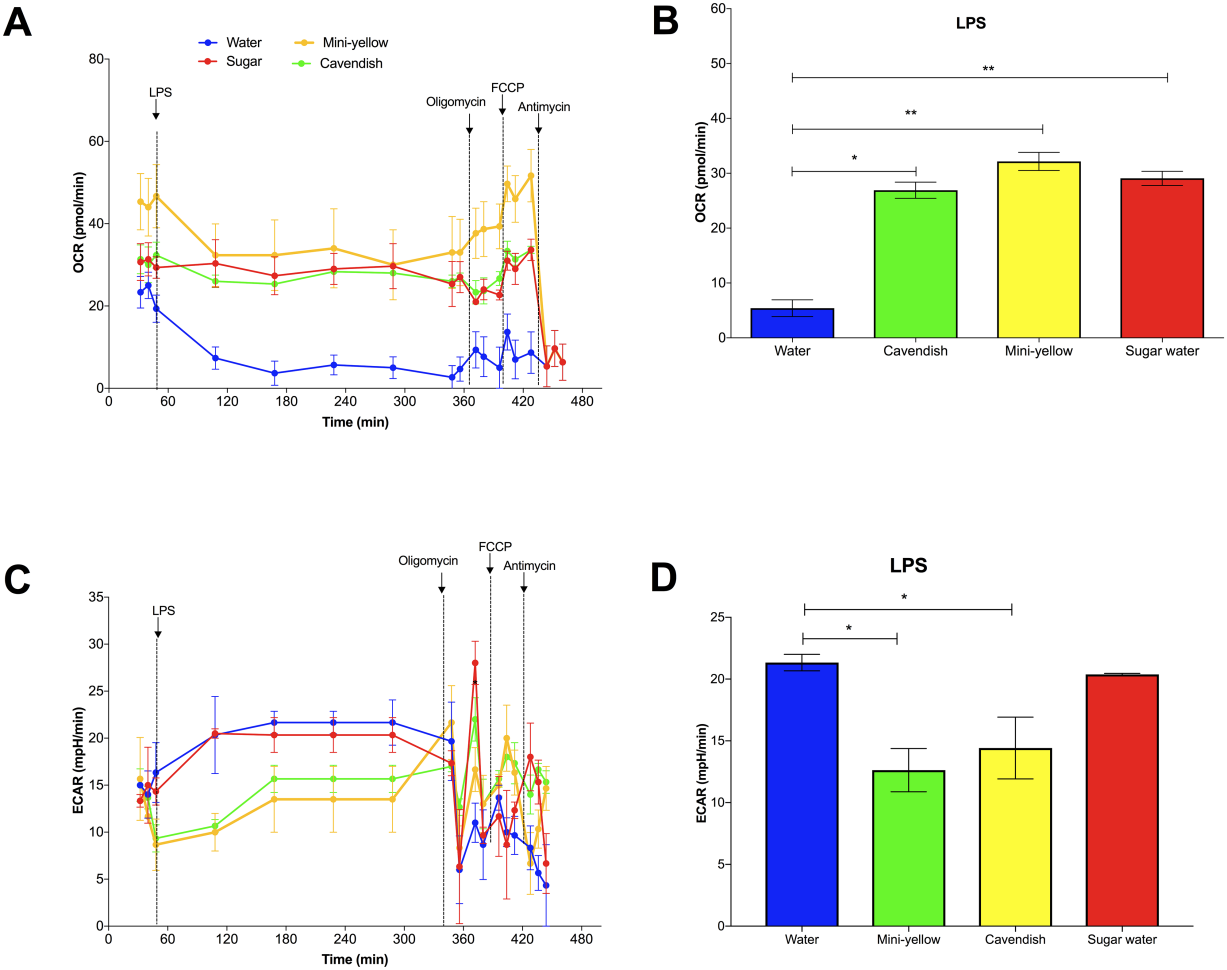
A) Changes in oxygen consumption rate (OCR) (basal state, after LPS stimulation, after injection of mitochondrial complex inhibitors) in THP1 monocytes immediately post 75-km cycling across four treatments (water only, Cavendish and mini-yellow bananas, and 6% sugar beverage) in cyclists. The bar chart (B) summarizes the LPS phase for OCR. C) Changes in extracellular acidification rate (ECAR) (basal state, after LPS stimulation, after injection of mitochondrial complex inhibitors) in THP1 monocytes immediately post 75-km cycling across four treatments (water only, Cavendish and mini-yellow bananas, and 6% sugar beverage) in cyclists. The bar chart (D) summarizes the LPS phase for ECAR. Data are mean ± SE. * Significantly different from the water-only trial, P≤0.05, respectively.

Following a 6-h incubation in plasma samples collected immediately post-exercise, monocyte OCR after LPS stimulation and following treatment with the mitochondrial complex inhibitors oligomycin and FCCP was significantly lower in the water-only trial compared to both of the banana and carbohydrate beverage trials (Fig 10A and 10B). Spare respiratory capacity [(maximal respiration)/(basal respiration) x 100] was 37.9% in the water-only trial and significantly lower when compared to 99.3%, 97.5%, and 90.5% in the mini-yellow banana, Cavendish banana, and sugar beverage trials, respectively. ECAR was significantly lower in the two banana compared to water and sugar beverage trials after LPS stimulation (Fig 10C and 10D).

## Discussion

Bananas contain a unique mixture of sugars, nutrients, and bioactive compounds such as phenolics, biogenic amines, and carotenoids [1–6]. This study utilized a metabolomics-based approach supplemented with specific measures of inflammation to determine potential influences on metabolic recovery from 75-km cycling while ingesting two types of bananas, a 6% sugar beverage, or water only. Regardless of the carbohydrate source (bananas or the 6% sugar beverage), carbohydrate intake at a rate of 0.2 g/kg every 15 minutes was associated with higher post-exercise plasma glucose and fructose, reduced plasma cortisol levels, diminished perturbation in lipid-related metabolites, and lower inflammation as assessed by total leukocyte counts, 9+13 HODES, and plasma IL-6, IL-10, and IL-1ra. This finding is consistent with other studies showing that acute carbohydrate ingestion (30–60 grams carbohydrate per hour) during intense and prolonged exercise attenuates inflammation in part by increasing blood glucose and tissue glucose uptake leading to diminished activation of the central nervous system (CNS), reduced output of adrenocorticotrophic hormone (ACTH), cortisol, epinephrine, and growth hormone, lowered lipid mobilization and oxidation, and reduced cytokine mRNA expression and release from muscle tissue [8,16,17]. Stress hormones have an influence on genes that control the function of immune cells and their release of cytokines, and exercise-carbohydrate interactions help modulate signal transduction cascades [18,19].

In agreement with previous publications from our group, cycling intensely for 75-km while ingesting water in an overnight fasted state was linked to a 2-fold or greater increase in 109 metabolites, with the majority reflecting exercise-induced lipid metabolite mobilization and oxidation [7,8,20–23]. The complex lipid panel showed that 65% of metabolites decreasing post-exercise were triacylglyceride esters (S2 Table), reflecting the manifold increase in free fatty acid mobilization and oxidation. OPLS-DA analysis of the metabolomics data showed that metabolic perturbation for the water only trial was significantly greater and sustained than for each of the two banana and sugar beverage trials. All three carbohydrate sources when compared to the water only trial were associated with significant increases for fructose, sucrose, and glucose, and significant decreases for multiple metabolites from the lipid super-pathway, the amino acid isoleucine, and the ratio 2-hydroxybutyrate/2-hydroxyisobutyrate from the glutathione sub-pathway.

An important aim of this research project was to determine if increases in plasma levels of banana-related metabolites following acute banana ingestion conferred any metabolic advantage during two days of recovery from intensive exercise beyond those linked to carbohydrate intake [7,8]. Banana flesh contains many unique molecules including dopamine and serotonin, and their precursors tyrosine and tryptophan, respectively [1–8]. Serotonin and dopamine consumed from bananas do not appear to cross the blood—brain barrier, and potential bioactive effects in the periphery may include regulation of glucose and lipid homeostasis, and enhanced gastrointestinal function [24–26]. Most of these data, however, are based on animal studies, and the clinical relevance of ingesting serotonin and dopamine from bananas is undetermined in humans.

OPLS-DA analysis of immediate post-exercise metabolite shifts showed a significant separation of Cavendish and mini-yellow banana trials from both the water only and sugar beverage trials. This separation included metabolites with significant fold changes and high VIP scores from ingestion of both types of bananas compared to intake of water or carbohydrate beverage including dopamine 3-sulfate, dopamine 4-sulfate, and related tyrosine metabolites (3-methoxytyramine sulfate and tyramine-*O*-sulfate), and 5-hydroxyindoleacetate (5HIAA, the primary breakdown product of serotonin) and related tryptophan metabolites (5-bromotryptophan, indoleacetate, kynureine, and 3-indoxyl sulfate). Other contrasting banana-related metabolites measured in the post-exercise plasma samples included those classified as xenobiotics (2-isopropylmalate, 4-acetylphenol sulfate, 2,3-dihydroxyisovalerate), sulfated phenolics (vanillic alcohol sulfate, ferulic acid 4-sulfate, caffeic acid sulfate, eugenol sulfate), urea cycle metabolites (argininate, trans-4-hydroxyproline, 2-oxoarginine, and proline), a methionine derivative (S-methylmethionine), a histidine metabolite (4-imidazoleacetate), and a metabolite of vitamin B6 (pyridoxate). Many of these metabolites were identified in a previous study from our research group [8].

Some of these plasma metabolites had substantially greater fold increases with mini-yellow banana ingestion (5HIAA, vanillic alcohol sulfate, 4-acetylphenol sulfate) and others with Cavendish banana ingestion (2-isopropylmalate, S-methylmethionine, 4-imidazoleacetate, 2,3-dihydroxyisovalerate, eugenol sulfate). Despite these differences, OPLS-DA analysis showed no differences between banana types when comparing total metabolome shifts immediately post-exercise, or during 2-days of recovery.

COX-2 mRNA expression increases strongly following intensive cardiorespiratory and resistance exercise in a variety of cell types including peripheral blood mononuclear cells and muscle cells [27–31]. COX-2 is an inducible enzyme, is abundant in activated macrophages, muscle cells, and many other cells at sites of inflammation, and converts the essential fatty acid arachidonic acid to prostaglandin. Prostaglandins are involved in inflammation and also help regulate numerous processes by acting on an array of cells through several different types of prostaglandin receptors [30]. A novel finding from the current study was that COX-2 mRNA expression in THP-1 monocytes was lower when cultured in plasma samples collected 21-h post-exercise from both banana trials compared to the water-only or the sugar beverage trials. These data suggest that banana flesh metabolites that increase in human circulation following ingestion may confer anti-inflammatory effects within monocyte cells as evidenced by reduced COX-2 mRNA expression the morning following heavy exertion.

The specific banana metabolite(s) responsible for this effect is (are) currently unknown, and additional studies have been initiated to make this determination. In vitro studies with a variety of cells including monocytes and macrophages indicate that numerous plant phytochemicals inhibit COX-2 mRNA expression [32–34]. Ingestion of plant phytochemicals such as those found in bananas may represent an effective strategy to exert anti-inflammatory effects by inhibiting COX-2 mRNA expression, but more randomized human trials are needed using tissue samples. In the current study, increases in plasma banana metabolites were associated with decreases in ex vivo, plasma cultured monocyte COX-2 mRNA expression but were not linked to reductions in plasma inflammatory cytokines beyond the effect observed for carbohydrate intake. These data suggest that within an exercise context, banana metabolites may function similar to aspirin or ibuprofen that inhibit COX activity but do not function as cytokine receptor antagonists [34,35]. Markworth et al. [36] reported that ibuprofen reduced plasma levels of prostaglandins and related metabolites in athletes following resistance exercise presumably because of inhibition of COX-1 and COX-2 mRNA expression. A direct correlation between reduced COX mRNA expression and lowered plasma levels of prostaglandins when banana metabolites are present in cell cultures will be investigated in additional studies by our group.

Analysis of immediate post-exercise samples from the water-only trial showed a decrease in basal and LPS-stimulated THP-1 monocyte oxygen consumption rate (OCR) using an extracellular flux analyzer, an effect which was countered when THP-1 monocytes were cultured in plasma samples obtained from the two banana and sugar beverage trials. Furthermore, the increase in extracellular acidification rate (ECAR) in LPS-stimulated THP-1 monocytes from post-exercise plasma samples from the water-only and sugar beverage trials was countered when monocytes were cultured in samples from both banana trials. Inflammatory activation of monocytes by LPS induces a rapid non-mitochondrial consumption of oxygen by NADPH oxidase-2 (NOX-2) known as the pro-inflammatory oxidative burst [37]. ATP production in monocytes is based both on mitochondrial oxidative metabolism (OCR) and glycolysis in the cytosol (measured as lactate production, ECAR) [37,38]. Following LPS stimulation, monocytes have the capacity to increase ATP production by increasing mitochondrial respiration or switching to glycolysis.

Data from the current study indicate an abnormally low spare respiratory capacity of LPS-stimulated monocytes that lacked the capacity to launch a pro-inflammatory oxidative burst response when cultured in plasma samples obtained from overnight fasted, exercise-exhausted athletes drinking only water. This finding is consistent with the viewpoint that the pre-differentiated monocytes in circulation are a good sensor of metabolic stress [37,38]. This impairment in monocyte function was countered when athletes ingested carbohydrate from the two types of bananas or the sugar beverage, indicating that the higher plasma sugar levels was sufficient to maintain mitochondrial oxidative metabolism. Metabolic switching to the glycolytic pathway (ECAR) was inhibited with the two banana trials, suggesting that banana metabolites had a role in mediating these effects. Frisard et al. [39] reported that LPS exposure caused skeletal muscle cells to metabolically switch from mitochondrial respiration to glycolysis, an effect countered by antioxidants (N-acetylcysteine and catalase), indicating some role for reactive oxygen species. Several of the banana metabolites measured in the plasma of the cyclists post-exercise exert anti-oxidant activity including dopamine and the sulfated phenolics [40].

### Conclusions

Improvement of the rate of metabolic recovery from intensive and prolonged exercise through nutritional support is an active area of research endeavor [18,41]. This study confirms and extends the findings from previous investigations showing that the primary nutrition-based strategy to attenuate exercise-induced metabolic perturbation and inflammation is acute carbohydrate ingestion of either sugar beverages or banana fruit [7,8,16,17]. Fruit provides more than sugars, however, and this study using THP-1 monocytes supports the hypothesis that banana metabolites appearing in plasma after ingestion exert anti-inflammatory effects by decreasing COX-2 mRNA expression. Furthermore, THP-1 monocytes cultured in plasma samples from the banana versus water trials relied more on the accepted default pathway of mitochondrial respiration rather than the more metabolically expensive pathway of glycolysis for ATP production. Taken together, these data support the combined intake of sugars and phytochemicals from banana fruit by athletes during heavy exertion as an efficient strategy to improve metabolic recovery and diminish post-exercise inflammation at the cell level.

## Supporting information

S1 Protocol Human research protection application and protocol summary. (PDF)

S1 Checklist CONSORT checklist. (DOC)

S1 Table Identified metabolite list. (XLSX)

S2 Table VIP ranking, exercise related metabolites. (XLSX)

S3 Table VIP ranking, carbohydrate related metabolites. (XLSX)

S4 Table VIP ranking, banana related metabolites. (XLSX)

S5 Table VIP ranking pre-to-post exercise ratios. (XLSX)
